# Supplementary material for: Voltage-driven motion of nitrogen ions: a new paradigm for magneto-ionics
Source: Nat Commun. 2020 Nov 18;11:5871. doi: 10.1038/s41467-020-19758-x (PMC7676264; doi:10.1038/s41467-020-19758-x)
Supplement: Supplementary file 1 — Supplementary Information [file 41467_2020_19758_MOESM1_ESM.docx]

**Voltage-driven motion of nitrogen ions: a new paradigm for magneto-ionics**

Julius de Rojas^1^, Alberto Quintana^2^, Aitor Lopeandía^1^, Joaquín Salguero^3^, Beatriz Muñiz^3^, Fatima Ibrahim^4^, Mairbek Chshiev^4^, Aliona Nicolenco^1^, Maciej O. Liedke^5^, Maik Butterling^5^, Andreas Wagner^5^, Veronica Sireus^1^, Llibertat Abad^6^, Christopher J. Jensen^2^, Kai Liu^2^, Josep Nogués^7,8^, José L. Costa-Krämer^3^, Enric Menéndez^1*^ and Jordi Sort^1,8*^

^1^Departament de Física, Universitat Autònoma de Barcelona, E-08193 Cerdanyola del Vallès, Spain

^2^Department of Physics, Georgetown University, Washington, D.C. 20057, United States

^3^IMN-Instituto de Micro y Nanotecnología (CNM-CSIC), Isaac Newton 8, PTM, 28760 Tres Cantos, Madrid, Spain

^4^Univ. Grenoble Alpes, CEA, CNRS, Spintec, 38000 Grenoble, France

^5^Institute of Radiation Physics, Helmholtz-Zentrum Dresden – Rossendorf, Dresden 01328, Germany

^6^Institut de Microelectrònica de Barcelona, IMB-CNM (CSIC), Campus UAB, E-08193 Bellaterra, Spain

^7^Catalan Institute of Nanoscience and Nanotechnology (ICN2), CSIC and BIST, Campus UAB, Bellaterra, E-08193 Barcelona, Spain

^8^Institució Catalana de Recerca i Estudis Avançats (ICREA), Pg. Lluís Companys 23, E-08010 Barcelona, Spain

*Email: [enric.menendez@uab.cat](mailto:enric.menendez@uab.cat) (E. Menéndez), [jordi.sort@uab.cat](mailto:jordi.sort@uab.cat) (J. Sort)

**Supplementary Text**

**Structural, magnetic and transport characterization of as-deposited films**

The investigated heterostructures comprise (i) 85 nm Co_3_O_4_ and (ii) 85 nm CoN, grown onto 60 nm Cu/20 nm Ti/0.5 mm [100]-oriented Si. The *θ*/2*θ* X-ray diffraction (XRD) patterns of the as-prepared Co_3_O_4_ and CoN films (Fig. S1a) are consistent with the presence of textured and polycrystalline Fd$\overline{3}$m Co_3_O_4_ and F$\overline{4}$3m CoN phases. From the XRD peak positions of CoN, a slightly expanded lattice parameter is obtained (*a* = 4.403 Å, *i.e*., 3% larger than the tabulated value), suggesting the presence of interstitial nitrogen. In fact, the relative Co (49.2 %): N (50.8 %) atomic ratio, obtained by electron energy loss spectroscopy, indicates a small off-stoichiometry in the film’s composition. Using XRD Rietveld refinement, the crystallite size in both systems is estimated to be between 10–25 nm depending on the crystalline orientation. Moreover, high resolution transmission electron microscopy (TEM) was performed in the cross-sections of as-prepared CoN and Co_3_O_4_ films (Fig. S1b and S2). The fast Fourier transform (FFT) analysis of the TEM images is consistent with CoN. Similarly, the FFT analysis in Fig. S2 gives spots whose positions are consistent with Co_3_O_4_.

Importantly, the as-prepared Co_3_O_4_ and CoN films exhibit no appreciable ferromagnetic behavior (black loops in Figs. 1b and 1c, Fig. S3), in agreement with their room-temperature paramagnetic nature. Moreover, the resistivity values at room temperature of Co_3_O_4_ (19 Ω cm) and CoN (4.11 × 10^–4^ Ω cm) (Fig. S4) reveal their insulating and semiconductor/metallic character, respectively. This allows the layers to hold electric fields across them, which is a necessary condition for magneto-ionic phenomena.

**Supplementary figures and tables**

**Supplementary Fig. S1**


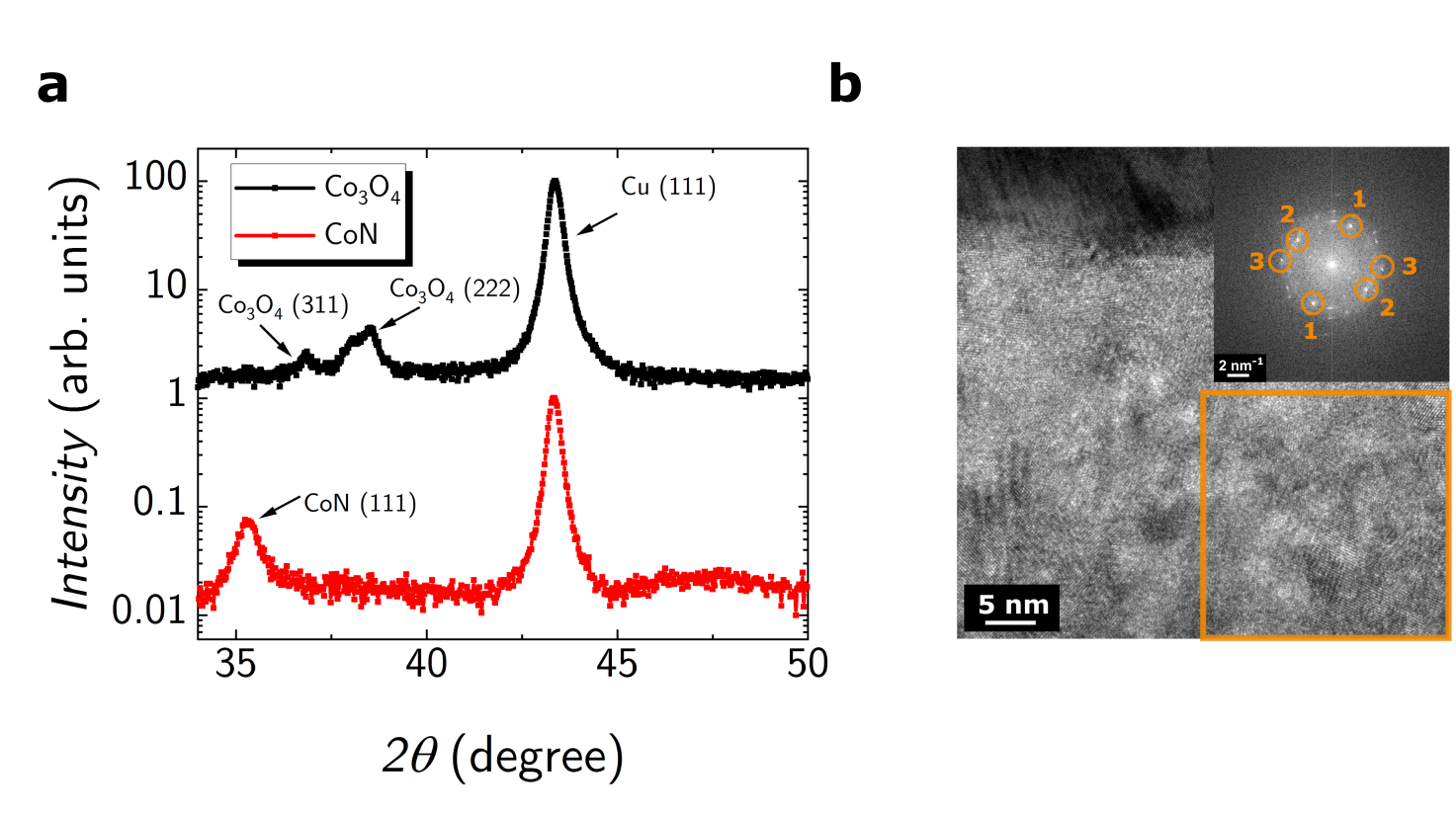


| ***Spot label*** | ***Interplanar distance d* (Å)** | ***(h k l) planes*** |
| --- | --- | --- |
| 1 (top right) | 2.56 | (1 1 1) |
| 1 (bottom left) | 2.56 | (1 1 1) |
| 2 (bottom right) | 2.59 | (1 1 1) |
| 2 (top left) | 2.60 | (1 1 1) |
| 3 (right) | 2.20 | (2 0 0) |
| 3 (left) | 2.21 | (2 0 0) |

**Fig. S1** **| Structural characterization by X-ray diffraction (XRD) and transmission electron microscopy (TEM). a** *θ*/2*θ* XRD diffraction patterns of the as-prepared Co_3_O_4_ and CoN films. **b** High resolution TEM image of the cross-section of an as-prepared CoN film. The inset shows the fast Fourier transform of the area marked with an orange rectangle. For phase identification, the cards no. ICDD JCPDF 00-009-0418 and ICDD JCPDF 00-016-0116, were taken for Co_3_O_4_ and CoN, respectively. The Table indicates the interplanar distances obtained from the spots of the fast Fourier transform in the inset of panel S1B. The corresponding Miller indices, according to the ICDD JCPDF 00-016-0116 CoN phase, are also given.

**Supplementary Fig. S2**

**
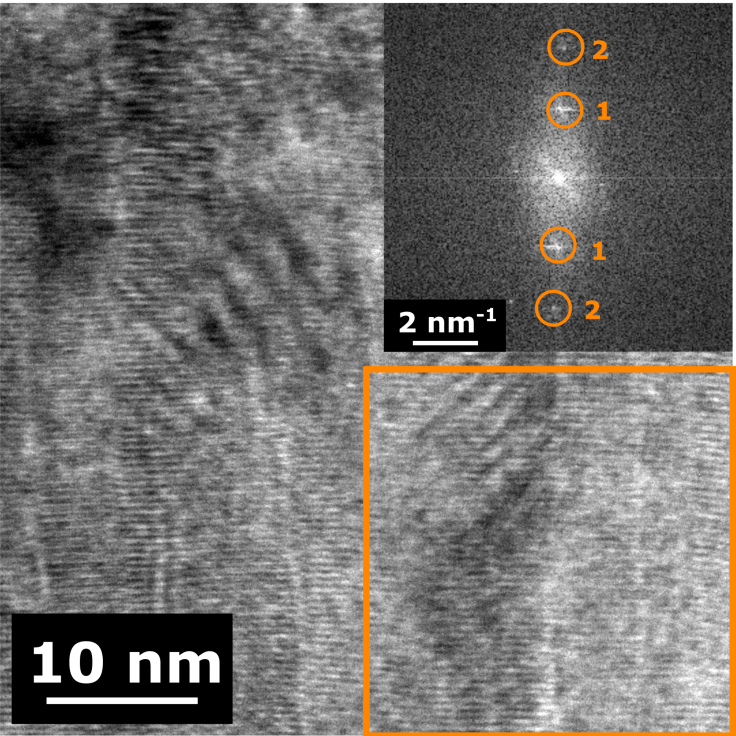
**

| ***Spot label*** | ***Interplanar distance d* (Å)** | ***(h k l) planes*** |
| --- | --- | --- |
| 1 (middle top) | 4.74 | (1 1 1) |
| 1 (middle bottom) | 4.68 | (1 1 1) |
| 2 (top) | 2.52 | (3 1 1) |
| 2 (bottom) | 2.51 | (3 1 1) |

**Fig. S2** **|** **Structural characterization by transmission electron microscopy (TEM).** High resolution TEM image of the cross-section of an as-prepared Co_3_O_4_ film. The upper inset show the fast Fourier transform of the area marked with a rectangle. The Table indicates the interplanar distances obtained from the spots of the fast Fourier transform in the inset of the figure. The corresponding Miller indices, according to the ICDD JCPDF 00-009-0418 Co_3_O_4_ phase, are also given.

**Supplementary Fig. S3**

**Fig. S3** **|** **Onset voltage and recovery process**. **a** and **b** consecutive hysteresis loops (each of 25 min of duration) under –6 V and –4 V gating and the corresponding recovery loops recorded at +6 V and +4 V for the Co_3_O_4_ and CoN films, respectively.

**Supplementary Fig. S4**





**Fig. S4** **|** **Electric transport properties.** Resistivity as a function of the temperature for the CoN film grown on an insulating substrate.

**Supplementary Fig. S5**





**Fig. S5** **|** **Saturation magnetization (*M_S_*) quantification from the hysteresis loops**. The schematic representation is based on the first hysteresis loop of the Co_3_O_4_ film upon electrolyte-gating at −50 V. All loops are previously slope-corrected for any linear contributions. For both descending and ascending branches of the loop (represented by arrows), *M_S_* is calculated in the negative and positive field regions above the anisotropy field: between 5 and 10 kOe in the positive field range, and −10 and −5 kOe in the negative field range. An average of the points is found as the effective *M_S_* (indicated with yellow dots): *M_1_*, *M_2_*, *M_3_* and *M_4_*, and linked to a time during the measurement of the loop. The time corresponding to this averaging region is half a minute.

**Supplementary Fig. S6**


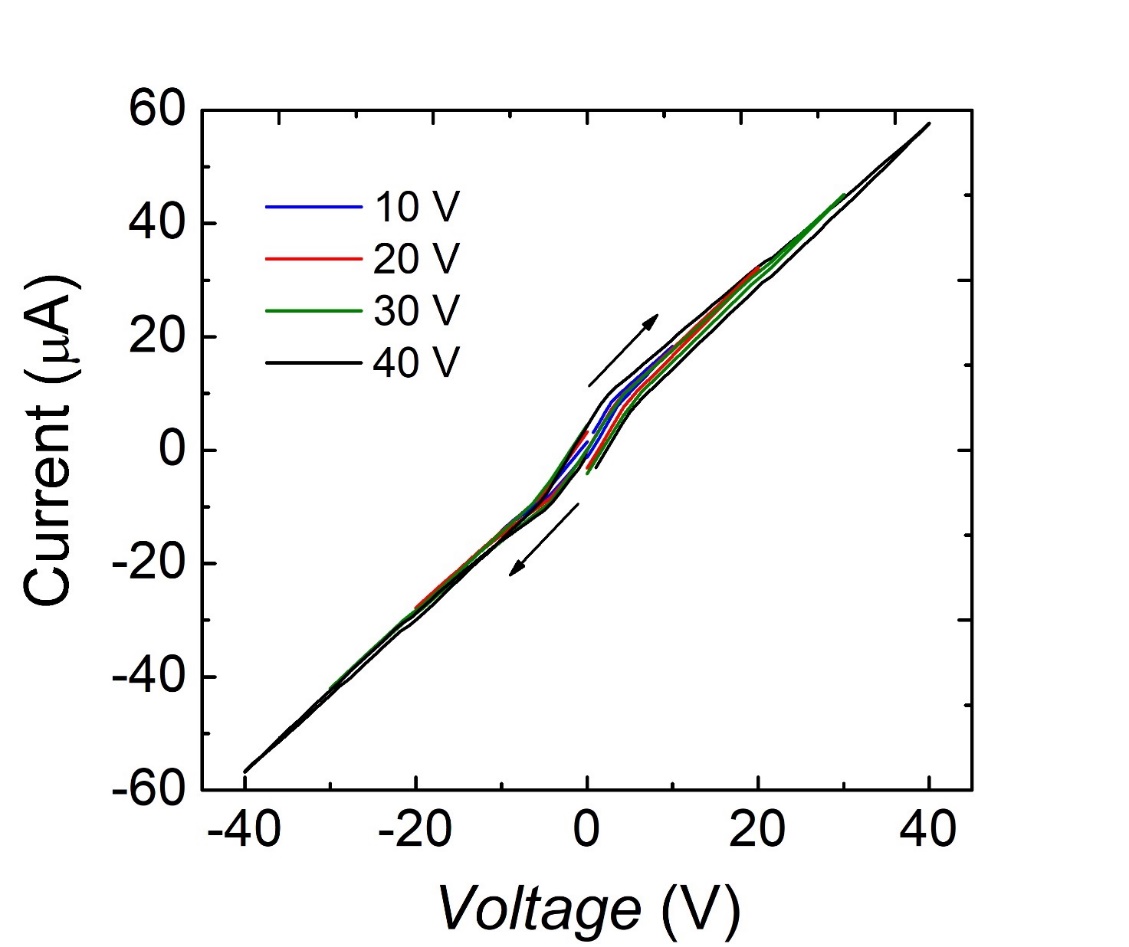


**Fig. S6|** **Cyclic voltammetry curves of Pt working electrode in anhydrous propylene carbonate with traces of Na^+^ and OH^–^ ions.** Cyclic voltammetry curves using different potential windows are shown. The curves were recorded at 250 mV/s with a Pt wire pseudo-reference electrode. The arrows indicate the direction in which potential was scanned.

**Supplementary Fig. S7**





**Fig. S7|** **Time evolution of the coercivity during electrolyte-gating**. Evolution of the coercivity (*H_C_*) with time for the Co_3_O_4_ and CoN films subjected to a voltage of−50 V for 12 hours. The inset represents zoom of the shaded region (first 90 min).

**Supplementary Fig. S8**

**
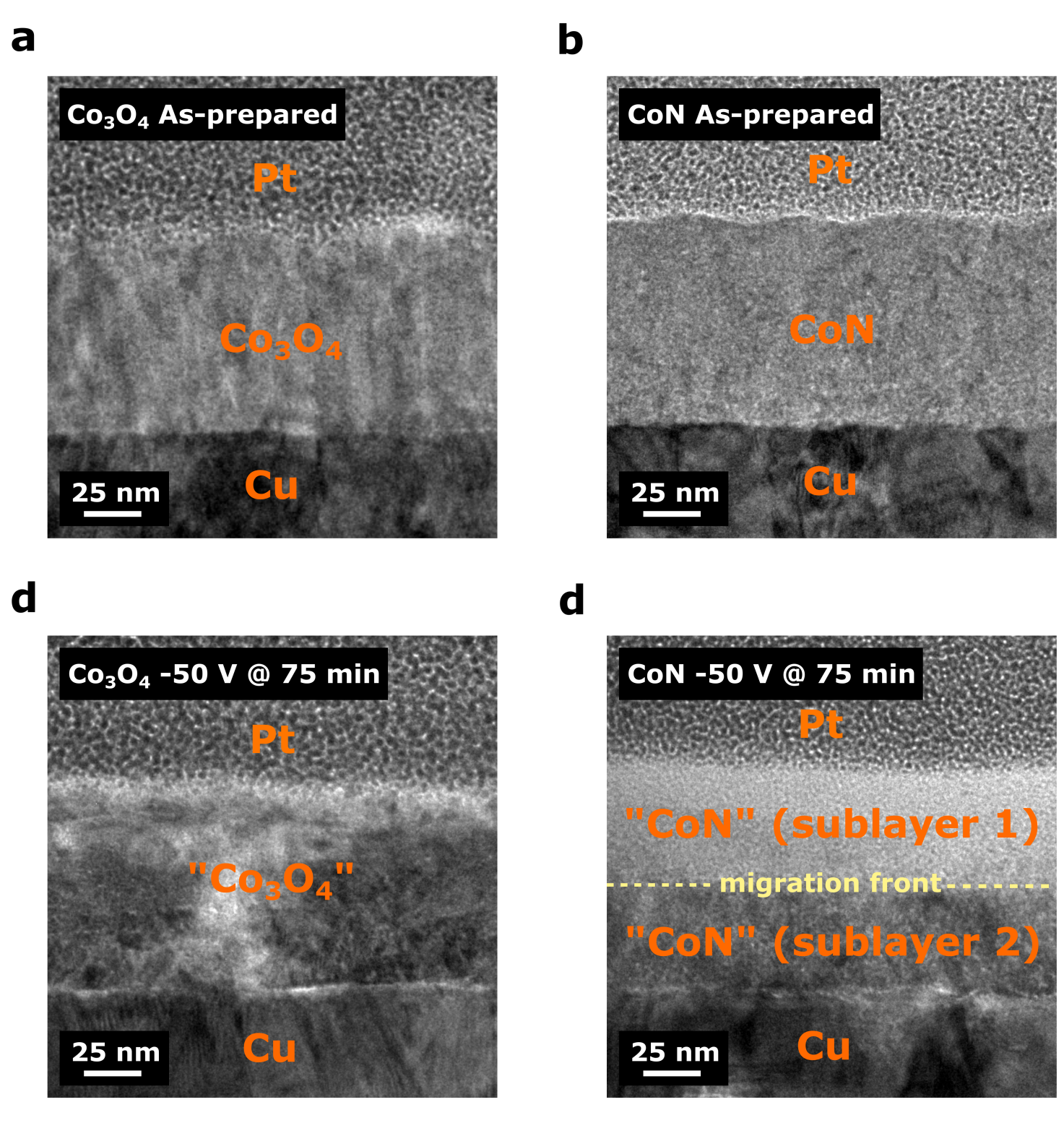
**

**Fig. S8** **|** **Structural characterization by transmission electron microscopy (TEM).** **a** and **b** are TEM images of the as-prepared Co_3_O_4_ and CoN films, respectively. **c** and **d** are TEM images of the Co_3_O_4_ and CoN films subjected to –50 V for 75 min, respectively.

**Supplementary Fig. S9**

**
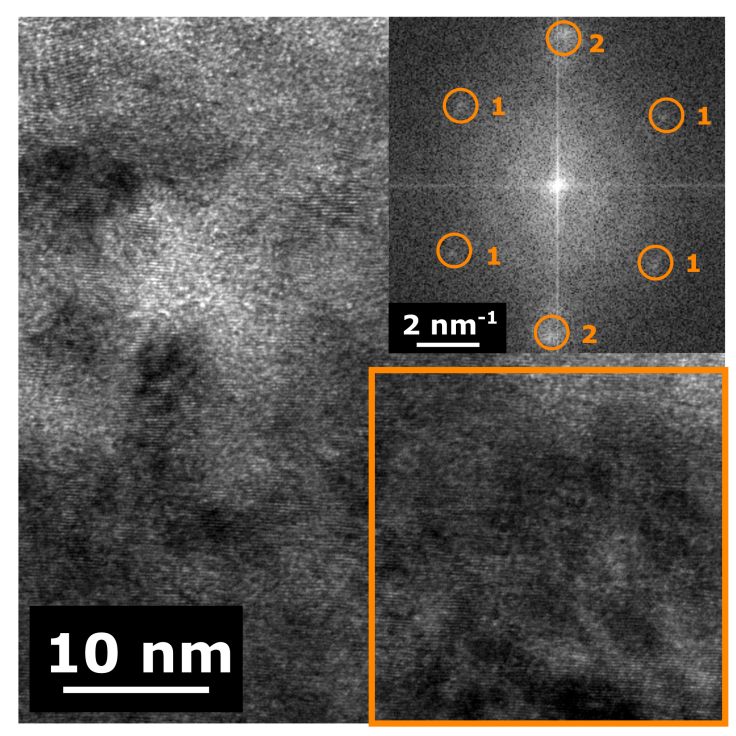
**

| ***Spot label*** | ***Interplanar distance d* (Å)** | ***(h k l) planes*** |
| --- | --- | --- |
| 1 (top right) | 2.51 | Co_3_O_4_ (3 1 1) |
| 1 (bottom right) | 2.48 | Co_3_O_4_ (3 1 1) |
| 1 (bottom left) | 2.52 | Co_3_O_4_ (3 1 1) |
| 1 (top left) | 2.51 | Co_3_O_4_ (3 1 1) |
| 2 (top) | 2.07 | Co_3_O_4_ (4 0 0)  HCP-Co (0 0 2)  FCC-Co (1 1 1) |
| 2 (bottom) | 2.06 | Co_3_O_4_ (4 0 0)  HCP-Co (0 0 2)  FCC-Co (1 1 1) |

**Fig. S9** **|** **Structural characterization by high resolution transmission electron microscopy (TEM) of the Co_3_O_4_ film after voltage actuation.** High resolution TEM image of the cross-section of a Co_3_O_4_ film treated −50 V for 75 min. The upper inset shows the fast Fourier transforms of the area marked with a rectangle. The Table indicates the interplanar distances obtained from the spots of the fast Fourier transform in the inset of the figure. The corresponding Miller indices, according to the ICDD JCPDF 00-009-0418 Co_3_O_4_, ICDD JCPDF 00-005-0727 hexagonal-closed packed (HCP) Co and PDF 00-015-0806 face-centered cubic (FCC) Co phases, are given.

**Supplementary Fig. S10**

**
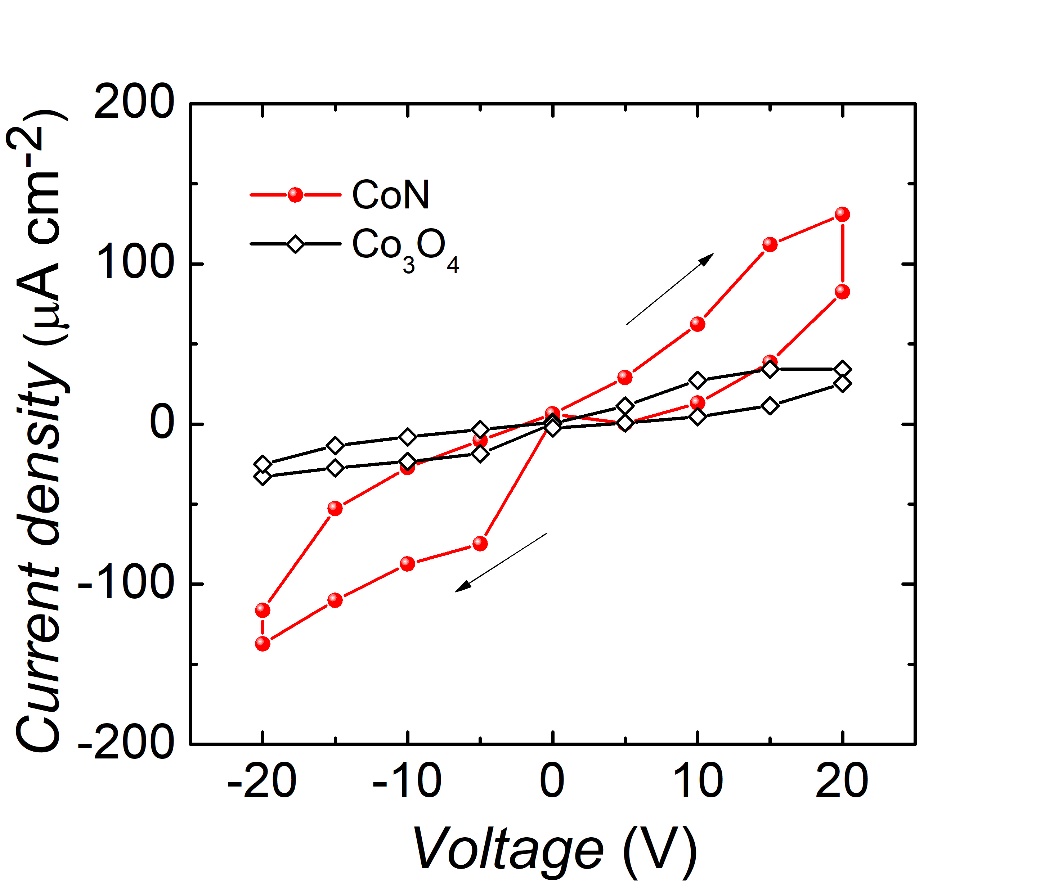
**

**Fig. S10|** **Cyclic voltammetry curves in anhydrous propylene carbonate with Na^+^ and OH^–^ ions for CoN and Co_3_O_4_ films.** The arrows indicate the direction in which potential was scanned.

**Supplementary Fig. S11**

**
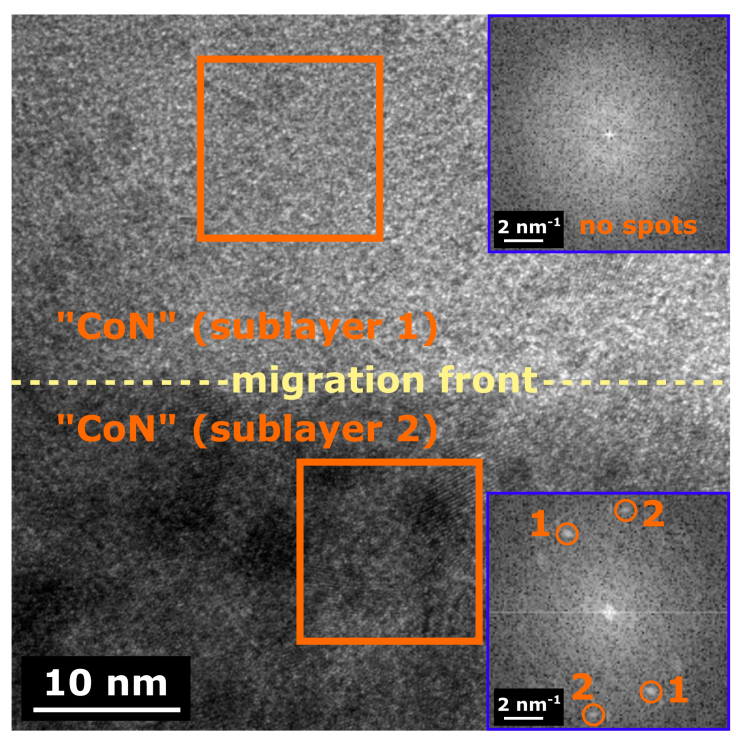
**

**Fig. S11** **|** **Structural characterization by high resolution transmission electron microscopy (TEM) of the CoN film after voltage actuation.** High resolution TEM image of the cross-section of a CoN film treated −50 V for 75 min. While clear diffraction spots can be seen in sublayer 2 (in contact with Cu), sublayer 1 (in contact with the electrolyte) is amorphous-like. Note that spots “2” likely correspond to HCP-Co (1 0 0) (ICDD JCPDF 00-005-0727), whereas spots “1” match the position of CoO (1 1 1) (ICDD JCPDF 00-001-1025). This oxide probably forms due to natural oxidation of the lamella (Co passivation).
